# Oxidation Resistance and Microstructure Evaluation of a Polymer Derived Ceramic (PDC) Composite Coating Applied onto Sintered Steel

**DOI:** 10.3390/ma12060914

**Published:** 2019-03-19

**Authors:** Tercius Justus, Priscila Gonçalves, Martin Seifert, Mateus L. Leite, Sônia M. H. Probst, Cristiano Binder, Günter Motz, Aloisio N. Klein

**Affiliations:** 1 Materials Laboratory (LabMat), Mechanical Engineering Department, Federal University of Santa Catarina, Florianópolis BR-88040-900, Brazil; tercius.justus@labmat.ufsc.br (T.J.); priscila.goncalves@labmat.ufsc.br (P.G.); sonia.probst@ufsc.br (S.M.H.P.); cristiano.binder@ufsc.br (C.B.); 2 Ceramic Materials Engineering (CME), University of Bayreuth, D-95440 Bayreuth, Germany; martin.seifert@uni-bayreuth.de (M.S.); Mateus.Lenz-Leite@uni-bayreuth.de (M.L.L.); guenter.motz@uni-bayreuth.de (G.M.)

**Keywords:** sintered steel, oxidation resistance, composite coating, polysilazane

## Abstract

Powder metallurgy is a competitive technology to produce ferrous near net shape parts for diverse engineering applications. However, their inherent porosity increases the susceptibility to oxidation and sealing their surface is mandatory to avoid premature degradation. Alongside, polymer derived ceramics (PDCs), such as silicon-carbonitride, have drawn attention concerning their high temperature and chemical stability. However, PDCs undergo volume shrinkage during ceramization that leads to defect formation. The shrinkage can be compensated by the addition of fillers, which are also capable of tailoring the ceramic resulting properties. This work evaluates the processing of PDC-based coatings loaded with ZrO_2_ and glass fillers to compensate for the shrinkage, densify the coating and seal the sintered steel surface. Therefore, polymeric slurries were sprayed onto sintered steel substrates, which were pyrolyzed at different temperatures for microstructural and oxidation resistance evaluation. Microstructural modifications caused by the enhanced glass viscous flow during pyrolysis at 800 °C resulted in more homogeneous, dense and protective coatings, which reduced the mass gain up to 40 wt% after 100 h of oxidation at 450 °C in air in comparison to the uncoated substrate. Moreover, no macrocracks or spallation were detected, confirming the feasibility of PDC composite barrier coatings for sintered steels.

## 1. Introduction

Increasing the lifetime of materials by improving specific and functional surface properties have fomented a millionaire growing market of engineered surfaces, due to the economic losses induced by deterioration of metallic parts [1], as reported by the latest National Association of Corrosion Engineers (NACE) international study on the global cost of corrosion [2]. The advancement of ferrous powder metallurgy (PM) in the last decades is closely related to its capability of providing an alternative processing technique at lower costs over other metal manufacturing technologies (e.g., forging, machining, stamping). Currently, the powder metallurgy industry meets the requirements from orthodontic, household appliances, hand tools to the automotive sector, which is responsible for almost 70% of the market [3,4,5,6]. This processing technology allows for microstructural and chemical composition control, besides economic benefits, especially in the case of high-volume production of small and complex geometries near net shape parts.

However, conventional sintered steel parts present inherent porosity and, according to the thermodynamic concept of surface energy, surfaces possess an excess of energy due to the lack of atomic bonds and achieve a lower state of energy reacting with atoms of the atmosphere. Therefore, the largest surface area of sintered steels makes them more susceptible to oxidation phenomena due to the increased active sites on the surface [7,8]. Although the mechanisms of wet corrosion, where the corrosive environment is composed of water with dissolved species, are different from the dry corrosion, in which the corrosive environment is a dry gas [9], their hindrance is dependent on the control and minimization of the surface porosity [10]. The common methods for pore sealing of sintered metallic parts range from steam treatments, impregnation with hydrophobic compounds and infiltration with low melting point alloys as well as mechanical methods, e.g., shot pinning processes. Furthermore, after pore sealing, the sintered metal part is usually coated to further enhance the corrosion resistance by methods as painting, electroplating, electroless deposition, hot-, dip- and spray-coating methods [5,10,11,12,13].

Recently, a new method for processing of silicon-based advanced ceramics was developed enabling a relevant technological advance in the manufacturing of ceramic fibers, coatings and ceramics by proper thermal treatment of polymeric precursors at lower temperatures (i.e., 500–1500 °C), compared with the classical ceramic powder processing technology. Polymer derived ceramics (PDCs) can be processed by conventional polymer shaping techniques, besides the capability of tailoring the final ceramic properties via microstructural and compositional design [14,15,16]. Polysilazane (PSZ), one class of ceramic precursor, generates SiCN ceramics, which, in turn, have potential application as protective coatings for ceramics and metal matrix composites, in situations where high-temperature stability, corrosion resistance or long-term durability need to be considered [17]. During thermal treatment, polysilazanes are initially cross-linked to form a non-meltable thermoset and then converted into an amorphous ceramic by the cleavage of chemical bonds, accompanied by the release of low molecular weight oligomers and gases (CH_4_, NH_3_) up to 800 °C under inert atmospheres [18,19].

The polymer to ceramic conversion induces a volume shrinkage of up to 70 vol%, generating residual stresses that can lead to defects, cracks and coating delamination, which limits the final coating thickness to only a few micrometers. To overcome such a drawback, the main strategy has been to load the preceramic polymer with suitable fillers to compensate for its shrinkage and tailor the coating final properties [14]. Passive fillers are considered inert in the system and do not undergo any chemical reaction nor mass or great volume change during pyrolysis. Examples of these fillers are usually ceramics with high-temperature stability such as SiC, Si_3_N_4_, ZrO_2_, TiO_2_, Al_2_O_3_ and SiO_2_ [20,21]. Active fillers react either with the precursor, its gaseous products or with the pyrolysis atmosphere to form new phases and compensate the shrinkage by expanding in volume. Thus, metals, semimetals, and silicides [22,23] are some commonly used materials. Meltable fillers, generally glasses [24,25], melt or soften during pyrolysis, sealing the coating porosity and reducing thermal stresses within it [14,26].

The combination of PDC and functional fillers provides versatile structural design and chemical composition to the final composite [27]. Powder metallurgy is largely used for producing ferrous net shape mechanical parts, which presents inherent porosity and increases the susceptibility to corrosion. Therefore, when exposing sintered steels to dry corrosion conditions (e.g., exhaust systems), the use of coatings with maximum sealing is of utmost importance to enhance their protective effect. This work evaluates the technical feasibility of applying a polysilazane-based/glass composite coating onto sintered steel to improve its oxidation resistance up to 450 °C in air, based on previous results reporting the enhancement on the oxidation resistance of low alloy steel sheets up to 700 °C in air by applying similar coating systems [24,25]. Nevertheless, in this study, the use of a perhydropolysilazane (PHPS) bond coating with outstanding oxidation resistance was not possible, considering its thickness limitation [21,25] and the intrinsic surface porosity of the used sintered steel substrates.

The coating system was developed from a commercially available oligosilazane precursor, loaded with ZrO_2_ to improve the coating oxidation resistance and reduce its thermal conductivity, besides glass fillers, used in order to offer viscous flow during thermal processing and promote the coating densification [24]. The chemical composition of the sintered steel used as the substrate allows the formation of martensite and bainite microstructures during the cooling step, enhancing its mechanical properties (97.4 wt% pre-alloyed Fe1.5Mo, 2 wt% pure nickel and 0.6 wt% graphite) [28,29]. Because of the lack of a PHPS bond-coat, and in order to avoid the oxidation of the metallic substrate during the pyrolysis of the coating, the thermal treatment was performed in nitrogen atmosphere.

## 2. Materials and Methods

### 2.1. Processing of the Sintered Steel Substrate

Cylindrical samples (Ø25 mm × 5 mm) were produced by the conventional powder metallurgy route, detailed in Table 1, using powder mixtures of 97.4 wt% pre-alloyed iron-molybdenum (Fe1.5Mo-Astaloy Mo, Höganäs, Halmstad, Sweden), 2 wt% of pure nickel (Epson Atmix PF-10F, d50 = 6.06 μm, Hachinohe, Japan) and 0.6 wt% of graphite (Micrograf 99511 UJ, Nacional de Grafite, d50 = 9.88 μm, São Paulo, Brazil). In order to assist the powder pressing, performed using a floating die and stationary lower punch, 0.8 wt% of particulate lubricant (Acrawax, Lonza, Basel, Switzerland) was added to the mixture. Sintering was carried out under a controlled atmosphere of 95% argon and 5% hydrogen (0.4 L min^−1^) with a heating rate of 5 K min^−1^. An average cooling rate of approximately 25 K min^−1^ was calculated from thermal data acquired during cooling until 430 °C, imposed by the gaseous flow and forced ventilation. The densities of the green and sintered steel samples were characterized by the geometrical method and compared with the theoretical densities, obtained by the rule of mixture.

### 2.2. Development of the Composite Barrier Coating

**Figure 1:**
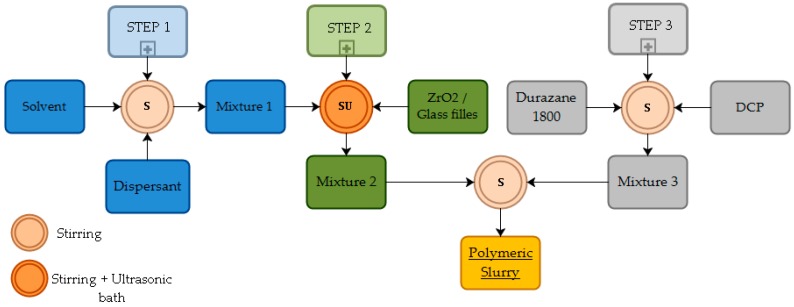
Flowchart with the step sequence for the processing of the polymeric coating slurry.

The polysilazane (PDC) filled composite barrier coating, with the composition presented in Table 2, was applied onto the sintered steel surface by spray-coating. Therefore, a homogeneous and stable precursor slurry was obtained following the scheme presented in Figure 1.

On the first step, 5 wt% of a dispersing agent Dysperbyk 2070 (BYK-Chemie GmbH, Wesel, Germany) regarding the amount of fillers was dissolved in di-n-butyl-ether (Acros Organics BVBA, Geel, Belgium) to assist the slurry formation. In sequence, the filler powders were added to the solvent in the respective proportions listed in Table 2, followed by the addition of Durazane 1800 and 3 wt% dicumyl peroxide (DCP) (Sigma-Aldrich Co. LLC., Darmstadt, Germany), regarding the amount of the polysilazane (step 3). DCP promotes the cross-linking via the double bonds and hydrosilylation of Durazane 1800 at temperatures above 130 °C, increasing its mass yield upon pyrolysis [30]. In order to avoid the formation of agglomerates, zirconia beads (Ø1 mm) and ultrasonic bath were used during the homogenization of the slurries (step 2). After coating the sintered steel substrates, pyrolysis was performed in a RO 10/100 tubular oven (Heraeus GmbH, Germany) at the temperatures of 700, 750, 770 and 800 °C, during 1 h in nitrogen atmosphere and using heating and cooling rates of 3 K min^−1^, based on the glasses vitreous and softening points [24]. 

### 2.3. Characterization Methodology

In order to determine the coating minimum thickness, the surface topography of the sintered steel samples was analyzed with an optical interferometer (Zygo Corporation, Newview 7300, Middlefield, CT, USA) and the topographic parameters Sa, Sq, Sp, Sv, Sz, Ssk, and Sku were obtained [31,32,33] by using the MountaisMap software^®^ (version 7.1.724, Besançon, Doubs, France). After spraying and pyrolysis of the steel substrates, the resulting coating thickness was magnetically measured using a Helmut Fischerscope (Sindelfingen, Germany) according to standard procedure ASTM B499-09 (2002).

For microstructural analyses, the cross-section of coated and uncoated substrates was obtained in a sequence of cutting, embedding and grinding the samples with SiC papers, followed by polishing with alumina suspensions of 1 and 0.3 μm and by etching with a solution of Nital 2%. The steel substrate, the coating and their interface microstructural characterization were performed by using light microscopy (Olympus BX 60, Hachioji, Japan and Zeiss Axiotech 100HD, Jena, Germany) and scanning electron microscopy (SEM) (JEOL JSM6390LV, Tokyo, Japan), equipped with an energy-dispersive spectrometer (Tescan Vega 3, Kohoutovice, Czech Republic). The evaluation of the microconstituents present in the sintered steel substrate was carried out by Vickers microhardness indentation (HV) using a load of 10 g during 10 s (Leco LM100AT, Saint Joseph, MN, USA). The crystalline phases were characterized by a Bragg–Brentano X-ray diffractometry (Bruker AXSD8, Karlsruhe, Germany and Philips Panalytical X’Pert MPD, Almelo, the Netherlands) with an energy of 40 kV and 40 mA, step size of 0.01° and 0.5 s of step time. The diffractograms were analyzed using the X’Pert High Score plus software (Almelo, Overijssel, the Netherlands) and the PDF 2+ database (2004).

It is important to highlight that, for microstructural analysis only, one face of the cylindrical steel sample was coated, whereas all free surfaces of the sintered steel were coated for the oxidation tests. Prior to these tests, the barrier property of the pyrolyzed coatings was qualitatively evaluated, based on the formation of a blue color known as the Prussian Blue. The objective was to determine the pyrolysis temperature range capable of effectively sealing the sintered steel surface. According to this method, a drop of HCl solution (5M) was applied onto the coated surface, followed by dripping potassium ferricyanide onto the same place. When potassium ferricyanide reacts with iron ions (Fe^2+^), a blue pigment is formed [34], which is an indication of whether the coating is acting as a barrier or not. Based on these results, cyclic oxidation tests were performed with fully coated samples in a chamber furnace (Nabertherm N41/H, Lilienthal, Germany) at 450 °C in air and different holding times as detailed in Table 3. Firstly, the furnace was heated up to the desired temperature and then loaded with the samples. After each specified holding time, the samples were removed from the furnace, allowed to cool down to room temperature, weighted and placed back into it. Three different oxidation tests were carried out up to 100 h, containing at least three samples of each system. The resulting average mass changes are reported within this work. For a matter of comparison, uncoated steel substrates were also placed into the furnace.

## 3. Results and Discussion

### 3.1. Sintered Steel

**Figure 2:**
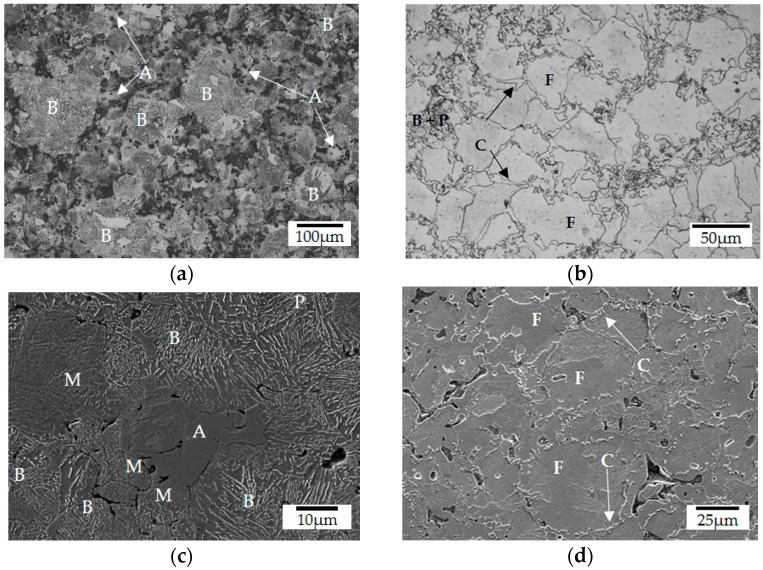
Typical sintered steel microstructure with indicated microconstituents: (**a**) optical and (**c**) secondary electron (SE) SEM micrographs after sintering (1150 °C); (**b**) optical and (**d**) SEM (SE) micrographs after pyrolysis (800 °C).

Densification of the steel substrate occurred mostly during compaction (89.1%) when compared with the theoretical density of 7.896 g cm^−3^, enhanced to 89.4% after sintering. Lourenço et al. reported that the surface porosity of steels sintered in conventional furnaces can range from 17% to 23%, depending on the sintering temperature [35], which is significantly higher than the 10.6% of porosity measured for the sintered steel sample volume. After sintering, the substrate presented a heterogeneous microstructure due to the composition of the powders used in the processing of the steel substrates in addition to the thermal treatment, i.e., final sintering temperature, dwell time and cooling rate. The use of a pre-alloyed iron-molybdenum powder as the main constituent and elementary nickel as alloying element increased the hardenability of the steel [28,36,37]. The cooling rate (~25 K min^−1^) of the sinter-hardening process promoted the formation of mostly bainite (B) microconstituent within the primary Fe1.5Mo particles, as indicated in Figure 2a. In addition, the use of elementary nickel promoted the formation of retained austenite (A) within the Ni particles, whereas martensite (M) was formed at its edges, where the concentration of nickel is reduced due to the diffusion phenomena. A mixture of bainite and pearlite (P) was detected within the interdiffusion regions in between the primary powder particles, as indicated in Figure 2c [38].

The sequential thermal treatments of sintering and pyrolysis led to a significant microstructural modification of the steel, as can be observed comparing Figure 2a,c to Figure 2b,d. During the pyrolysis of the coating performed at lower temperatures (up to 800 °C) and heating and cooling rates (3 K min^−1^), a microstructure transformation to a mixture of mainly ferrite (F), pearlite (P) and cementite (C) at the grain boundaries was observed. This occurred due to the carbon reduction in the eutectoid point, which favors free carbide formation, induced by the presence of Mo and Ni in the steel matrix [39]. This phenomenon is associated with the lower treatment temperatures, which were not able to completely austenitize the iron-based matrix. Moreover, in the spots where austenitization occurred, bainite and pearlite were formed.

These results were confirmed by Vickers microhardness and EDS analyses (energy dispersive spectrometry), which are summarized in Table 4 and are in good agreement with values reported in the literature [40]. The highest standard deviation of the measurements observed after sintering are associated with the formation of martensite on edges of the retained austenite (±107 HV) at the nickel-rich regions, and at the cementite phase (±113 HV) due to the difficulty of indentation precisely on the spot, located at narrow regions. It is important to highlight that the precipitation of carbides at steel grain boundaries must be avoided, since it can lead to intergranular failure, worsening the mechanical properties of the steel [41,42].

**Figure 3:**
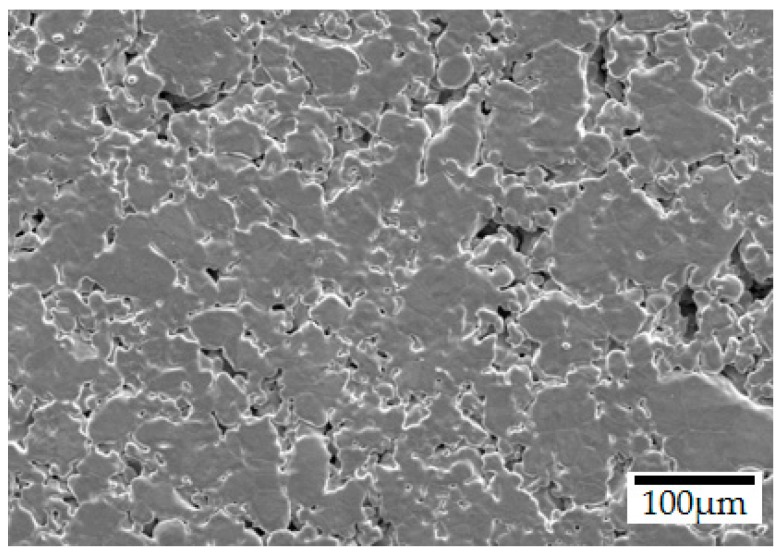
Typical SEM surface micrograph (SE) of the steel after sintering.

Topographic parameters obtained by optical interferometry are presented in Table 5, together with a representative SEM surface micrograph of the sintered steel in Figure 3. The negative value of skewness (Ssk) combined with a Kurtosis (Sku) value higher than 3, indicates a surface containing high peaks and deep valleys, confirmed by the parameters maximum peak height (Sp), maximum valley depth (Sv) and maximum height of the surface (Sz), as already reported [31,32,43,44]. However, the sintered steel surface is mostly flat, indicated by the low arithmetical mean height (Sa) and shown in the SEM surface micrograph. This aspect was promoted mainly during the powder compaction step. According to a statistical concept named the coefficient of variation (CV) [45], the calculated CV value of 155% indicates that the sintered steel does not possess a homogenous surface, by using the arithmetical mean height (Sa) and the root mean square roughness (Sq) as standard deviation. This interpretation is corroborated when comparing the maximum height of the surface (Sz) with the arithmetical mean height (Sa) measured. Lastly, the mean maximum height (Sz) result indicates that the height difference between a valley and a peak of the surface can reach up to 34.60 ± 3.62 µm, and, therefore, a coating thickness of 38 µm was aimed to fully coat and protect the surface.

### 3.2. Composite Barrier Coating onto the Sintered Steel Substrate

**Figure 4:**
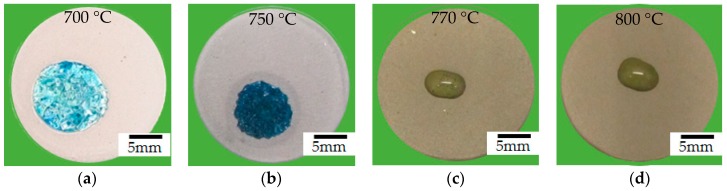
Drops and appearance of the blue color onto coated samples pyrolyzed at: (**a**) 700 °C; (**b**) 750 °C; (**c**) 770 °C and; (**d**) 800 °C, 1 h in N_2_ atmosphere.

After coating the sintered steel with the polymeric slurry and pyrolyzing sets of samples at 700, 750, 770 and 800 °C, the average coating thickness achieved was 34 ± 7 µm due to slight speed variations during manual spraying. Regarding the Prussian Blue evaluation performed, the appearance of a blue color after applying a drop of potassium ferricyanide at the acid solution etch, presented in Figure 4, indicates a chemical reaction with the steel. The results pronounce the influence of the pyrolysis temperature on the protective effect of the coating. When performed above the glass softening point of both fillers, borosilicate (570 °C) and barium silicate (770 °C), the sealing of the composite coatings and its associated barrier properties were improved.

**Figure 5:**
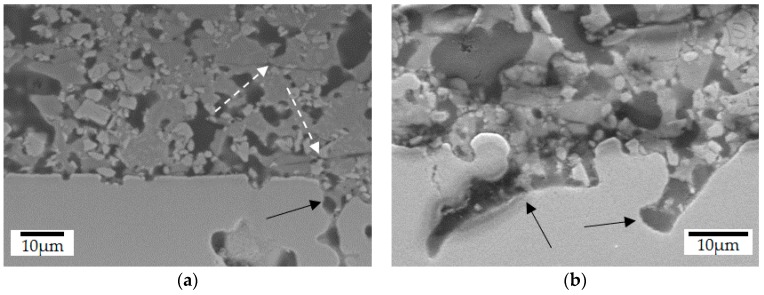
Interface cross-section of coated sintered steel, pyrolyzed at 800 °C during 1 h in N_2_ atmosphere: (**a**) general aspect of the coating; (**b**) surface pore filling in detail (SEM/SE).

**Figure 6:**
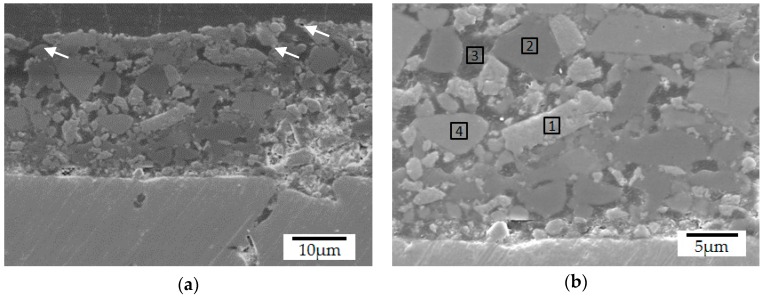
Typical coating cross-section after pyrolysis at 700 °C, during 1 h in N_2_ atmosphere: (**a**) open surface pores; (**b**) main microconstituents (SEM/SE).

The qualitative evaluation of the interface revealed a good infiltration of the coatings in the sintered steel as well as the filling of the surface porosity, as shown in different magnifications in Figure 5 and Figure 6. Although pyrolysis at 700 °C was not enough to seal the coating and create a barrier, as previously indicated (Figure 4), this temperature already promoted the infiltration of the substrate surface. Enamel coatings are known to adhere to steels due to mechanical anchoring into the surface roughness [46,47,48], an effect which can be further increased in sintered steels due to their intrinsic porosity. Furthermore, the absence of cracks between the coating and the steel substrate indicates good adhesion, while the presence of cohesive cracks in the coating, indicated by the white arrows in Figure 5a, supports that this class of PDC barrier coating fails by cohesive rather than by adhesive mechanisms [25]. 

Further microstructural analyses of samples pyrolyzed at lower temperatures (<770 °C) indicated that the Prussian Blue test results may be related to the open porosity observed in these composites, as shown in Figure 6a. The coating microstructure is composed of four main constituents, the original filler particles and porosity, indicated in Figure 6b, aggregated by the amorphous PDC phase. Concerning the fillers’ chemical composition and SEM/EDS analysis, region 1 was associated with ZrO_2_ particles (59.0 wt% of Zr), whereas region 2 corresponds to the boron silicate glass (8.1 wt% of Na and 9.4 wt% of Zn) and region 4 to the barium silicate glass (55.0 wt% of Ba). Additionally, the dark grey sites (region 3) were characterized as the coating residual porosity, filled with embedding resin during the sample preparation. Due to the presence of common elements to the glass fillers and the PDC-phase and the difficult detection of light elements as C and N, no precise identification of this phase was possible by means of EDS-analysis (see Figure S1, Supplementary Materials).

**Figure 7:**
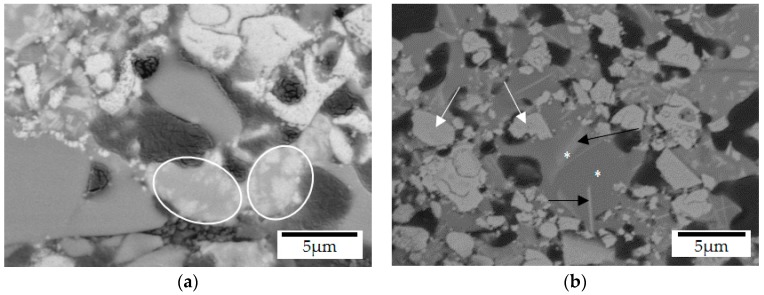
Glass fillers mixtures observed in the coating microstructures (SEM/BSE-backscattered electrons) after pyrolysis at (**a**) 750 °C and (**b**) 800 °C during 1 h under N_2_ atmosphere.

When the coating pyrolysis was performed at 750 °C, closer to the softening temperature of the barium silicate glass (770 °C), the reduced viscosity of both glasses promoted the mixing of their corresponding phases, as highlighted in Figure 7a [49,50]. As expected, after pyrolysis at 800 °C, an almost complete homogenization of the glasses was observed and only traces of the original barium silicate glass were detected (narrow white regions in Figure 7b indicated by the black arrows). EDS analysis performed in these white regions and in the surrounding glass matrix, indicated with stars, revealed similar amounts of sodium (3.7 and 4.7 wt%) and barium (35.7 and 26.5 wt%). Despite the higher densification of the composite coating at 800 °C, closed residual porosity is still present at its surface (see Figure S2, Supplementary Materials, and Figure 4d). Due to the higher thermal stability of ZrO_2_, no changes in its microstructure or composition were detected by SEM/EDS-analysis after pyrolysis, as indicated by the white arrows in Figure 7b and supported by Figure S1.

The coating microstructures achieved in this work presented differences regarding the chemical composition and the resulting porosity, in comparison with reported by Günthner et al., where denser and homogeneous coatings were obtained even at a lower pyrolysis temperature in air (700 °C) [24]. The dissimilarities observed were attributed to two main factors: (1) pyrolysis atmosphere and (2) the use of a sintered steel substrate.

The precursor Durazane 1800 contains reactive Si-N and Si-H bonds, known by their sensitivity to air and moisture [18,51]. Depending on the pyrolysis atmosphere, ceramics with distinct elemental composition are generated, in regard to the amount of carbon, nitrogen, and oxygen. Its pyrolysis in air up to 1000 °C and using 3 wt% of DCP, results in a ceramic with 83% mass yield and the elemental composition of SiC_1.15_N_0.68_O_0.42_ [24,51,52], whereas pyrolysis in protective atmosphere results in a ceramic yield of 75% with the elemental composition of SiC_0.8_N_0.9_ [19,51,53]. The higher mass yield observed for pyrolysis in air is attributed to the increased cross-linking induced by the incorporation of oxygen in the precursor under the release of NH_3_ and H_2_, which explains the lower nitrogen content. In the performed experiments, the increased nitrogen partial pressure reduced the elimination of ammonia and the incorporation of oxygen, which led to a lower degree of cross-linking. Thus, upon heating a greater evolution of volatile oligomers and hydrocarbons is observed, causing a lower mass yield and carbon content [18,51]. Anyhow, in comparison to the ceramic composition after pyrolysis in nitrogen atmosphere, it is expected that the coatings produced in this work were also influenced by oxygen since the handling and spraying of the steel samples were carried out in air.

Another reason for the differences observed can be attributed to the porosity of the sintered steel substrate. During pyrolysis, the glass fillers and oligosilazane infiltrated its surface as made evident in Figure 5, thus enhancing the formation of porosity within the coatings (Figure 6a). After infiltration and sealing of the substrate surface, the better glass flow promoted at higher pyrolysis temperatures (770 and 800 °C) due to the reduced viscosity enabled the coating densification. Thus, the effects associated to the pyrolysis of Durazane 1800 in nitrogen atmosphere and the porosity of the steel substrate are possibly the reasons for the increased porosity and inhomogeneous microstructure observed in these coatings.

Taking into account the coating enhanced sealing effects after pyrolysis above 750 °C in N_2_ atmosphere, the samples for the oxidation tests were pyrolyzed at 770 °C and 800 °C, following the same pyrolysis procedure. The tests with the coated samples after pyrolysis at 770 °C were carried out in attempt to reduce the coating processing temperature to a lowest possible and still protect the sintered steel during cyclic oxidation. The oxidation temperature was set to 450 °C, based on the lower vitreous transition temperature of the boron silicate glass and on the fact that low alloy carbon steels, which form surface oxides, are commonly used up to 500 °C [54]. It is well known that iron and low alloy carbon steels oxidized up to 570 °C form two oxide layers, a thicker inner layer of magnetite (Fe_3_O_4_) and a thin outer layer of hematite (Fe_2_O_3_) [54,55,56]. Bertrand et al. further observed that this oxide scale, formed in both dry and wet air (2 vol% H_2_O) and in the temperature range from 260 to 400 °C, is composed of three different microstructures [57]. A very thin layer of small columnar grains at the metal/oxide interface (magnetite), a thicker layer of coarser columnar grains (magnetite) above it and at the oxide/atmosphere interface a very thin layer of equiaxed grains (hematite). The oxide growth mechanism proposed by the author states that the external part of the magnetite and the hematite layers grow by cation outward diffusion, while cation vacancies diffuse inward the magnetite layers. Hematite growth is controlled by interstitial cation and grain boundary diffusion, therefore, the rate-controlling step of oxidation kinetics is the cationic diffusion in the external magnetite layer, which is the thickest of the three layers and has the largest grain size, presenting a parabolic oxide-growing rate.

**Figure 8:**
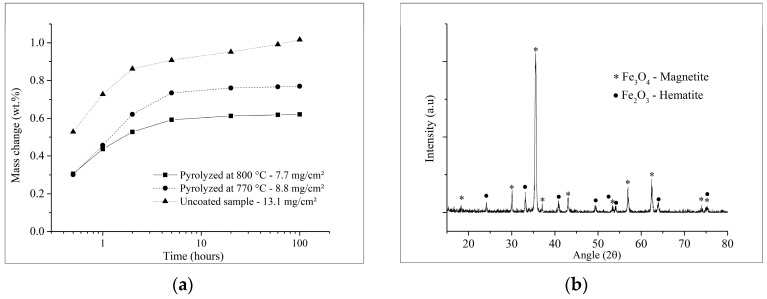
Oxidation results after 100 h at 450 °C: (**a**) uncoated and coated sintered steel mass change; (**b**) typical X-ray diffraction pattern of the uncoated steel after the oxidation test.

The results of the oxidation tests, of coated and uncoated samples plotted in Figure 8a, indicate the mass change percentage with treatment time, and the X-ray diffractogram obtained from the oxidized sintered steel in Figure 8b. Uncoated samples presented a mass gain of 13.1 mg cm^−2^ after 100 h at 450 °C, with the formation of both hematite and magnetite iron oxides on its surface. Moreover, considering the cation–diffusion growth mechanism of the oxides layers cited above, as well as the oxidation kinetics and X-ray results, it is possible to conclude that uncoated sintered samples presented a parabolic oxidation rate. As it can be observed in Figure 8a, there is an initial transitory period of faster kinetics of oxidation followed by a parabolic behavior, controlled by diffusion and limited by the growing oxide scale, as corroborated by results found in the literature for sintered and wrought iron alloys [57,58,59,60].

In contrast, coated samples pyrolyzed at 770 and 800 °C after the same oxidation time presented an average mass gain of 8.8 and 7.7 mg cm^−2^, which are respectively 33 and 41% lower than the measured for the uncoated steel. Moreover, the oxidation behavior of the coated and uncoated samples was similar up to 20 h of testing; nevertheless, the mass gain of the coated samples was, respectively, 22 and 45% lower than the uncoated sintered steel for this time. The tendency of a reduced mass gain of the coated samples after 20 h is remarkable, as demonstrated in Figure 8a, which resembles the oxidation behavior of passivating metals at low temperatures and follows logarithmic kinetics [54].

In order to quantify such oxidation behavior, the parabolic rate constant (k_p_) was calculated from the mass gain measurements by using the classical law for parabolic oxidation kinetics (Equation (1)), also used by Trindade et al. [59], despite its known limitations [61], in which Δ*m* is the mass-gain (mg), *A* is the apparent surface area (cm^2^) and *t* is time (s):

(1) $${\left(\frac{\Delta m}{A}\right)}^{2}=k_{p}t.$$

**Figure 9:**
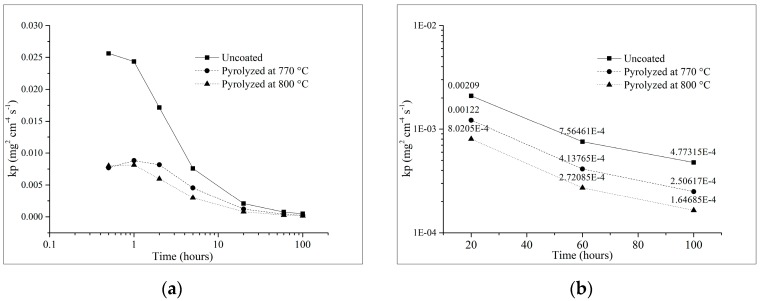
The apparent instantaneous parabolic rate at 450 °C, for uncoated and coated sintered steel; (**a**) up to 100 h of test; (**b**) detail of the values from 20 h of oxidation test.

The apparent instantaneous parabolic rate constant *k_p_* [61] plotted against the oxidation time in Figure 9a visually indicates the lower oxidation rates of the coated samples in comparison with the uncoated substrate during the first 5 h. Although the results suggest that the rates move towards similar values from 20 h on, it is possible to notice a separation of the curves in Figure 8a, while the mass gain of the uncoated steel further increases with time, that of the coated samples tend to stabilize. This deviation from the original parabolic kinetics of steel oxidation indicates a gradual decrease of the oxidation process, until passivation or completely stagnation is achieved, corroborated by the increasing difference in the oxidation rates (*k_p_*) between coated and uncoated samples, shown in Figure 9b. After 20 h of oxidation, the coated samples pyrolyzed at 770 and 800 °C presented oxidation rates of 41.6 and 61.7% lower than that for the uncoated samples, this difference increased respectively to 47.5 and 65.5% after 100 h of oxidation.

**Figure 10:**
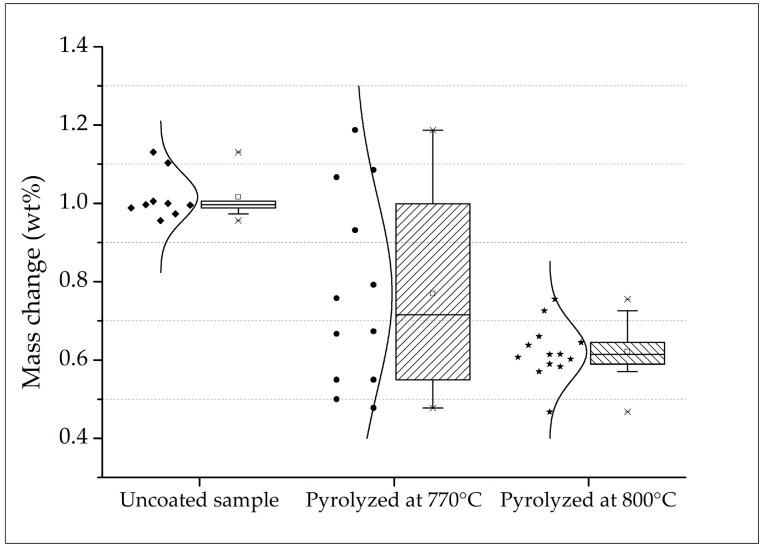
Boxplot with data distribution of measured mass gain after 100 h of cyclic oxidation.

Both sets of samples pyrolyzed at 770 and 800 °C presented no visible cracks or spallation after the cyclic oxidation tests, which is also an indicative of good adhesion of the coatings onto the sintered steel surface. Despite no statistical differences were indicated by the Student’s *t*-test hypothesis with 5% of significance for the coated samples up to 100 h of oxidation, the samples pyrolyzed at 770 °C presented a higher variability, as demonstrated in Figure 10. Accordingly, a high coefficient of variation was calculated for this set of samples (CV = 33%). In this case, the average mass change is not reliable [45,62]. In contrast, the coated samples pyrolyzed at a higher temperature (800 °C) presented a calculated CV of 11%, comparable to the uncoated steel substrate. Therefore, the resulting average mass change in these cases are more reliable. 

With exception of a few good results observed, the pyrolysis at 770 °C did not promote enough viscous flux of the barium silicate glass to seal the coating and protect the surface of many samples. This might be associated with slight temperature gradients in the tubular furnace, thus not effectively reaching the softening temperature of the glass (Ts = 770 °C), which can explain the high variability. Furthermore, the increased mass gain detected in the first 5 h of test must be better investigated, in attempt to further reduce the initial oxidation of the coated samples.

## 4. Conclusions

The achievement of coatings with an average thickness of 34 µm, capable of filling the pores on the sintered steel substrate surface and the absence of macro defects confirm the feasibility of processing PDC composite barrier coatings on such porous substrates. SEM/EDS analyses revealed no reaction between the coating systems and the steel substrate. The mechanical anchoring of the glassy coating within the surface of the steel substrate leads to an enhanced adhesion. Accordingly, no adhesive cracks were detected at the substrate-coating interface. In comparison to the uncoated substrate, the mass gain of the coated samples pyrolyzed at 770 and 800 °C presented decreased average values of, respectively, 33 and 41% after 100 h of oxidation at 450 °C in air. Anyhow, the enhanced glass flow at 800 °C led to the processing of more homogeneous, dense and protective coatings, in agreement with the lowest mass gain and coefficient of variation obtained at this temperature. 

As expected, the uncoated sintered steel samples presented a parabolic oxidation rate, whereas the coated samples presented both parabolic and logarithmic behavior. The first can be related to the transitory period of fast oxidation (first 5 h), shown for both coated and uncoated samples. The second, observed for the coated samples after 20 h, deviated from the oxidation behavior of the uncoated substrate, indicating gradual passivation or stagnation of the process, typical of logarithmic kinetics. This tendency is demonstrated by the relative difference between the oxidation rates after 20 h, which therefore suggests the efficiency of the PDC barrier coating to protect the sintered steel substrates against dry oxidation at the evaluated temperature. Nevertheless, slight adjustments in the pyrolysis temperature or in the coating or steel composition may contribute further to develop a more stable oxidation system and should avoid detrimental microstructural changes, such as iron carbide precipitation at the grain boundaries during pyrolysis.

## Figures and Tables

**Table 1:** Apparatus and processing parameters for the sintered steel production.

Processing Steps	Apparatus	Parameters
Mixing	Y-type mixer	45 min
Pressing	Hydraulic press	600 MPa
Lubricant-debinding	Tubular furnace	500 °C, 30 min
Sintering		1150 °C, 60 min

**Table 2:** Composite barrier coating composition before pyrolysis.

Material	Manufacturer	Description	vol%	d_50_ (µm)
Durazane 1800	Merck KGaA, Germany	Polymeric precursor	20.0	-
Glass 8470	Schott AG, Germany	Borosilicate glass	27.5	3.3
Glass G018-311		Barium silicate glass	27.5	3.1
ZrO_2_	Alfa Aesar GmbH & Co KG, Germany	Zirconium oxide	25.0	1.0

**Table 3:** Cyclic oxidation test parameters (450 °C, air).

Measurement No.	1	2	3	4	5	6	7	8
Oxidation time (h)	0	0.5	1	2	5	20	60	100

**Table 4:** Microhardness of microconstituents (HV) and EDS ^1^ composition analysis (wt%) after sintering and pyrolysis.

**After Sintering**	**(HV)**	**Fe**	**Ni**	**Mo**
Bainite	316 ± 44	96.0	0.1	1.2
Austenite/Martensite	393 ± 107	89.6	6.8	1.3
Perlite/Bainite	379 ± 46	95.5	0.5	1.3
**After Pyrolysis**	**(HV)**	**Fe**	**Ni**	**Mo**
Cementite	782 ± 113	89.6	2.1	1.6
Ferrite	162 ± 11	93.7	1.7	1.7
Perlite/Bainite	265 ± 34	89.7	-	1.8

^1^ Metallic elements.

**Table 5:** Average topographic parameters of the sintered steel.

Parameter	Acronym	Average	Standard Deviation
Arithmetical mean height	Sa	2.03 µm	0.13
Root mean square roughness	Sq	3.15 µm	0.21
Maximum peak height	Sp	11.25 µm	1.60
Maximum valley depth	Sv	23.37 µm	3.60
Maximum height of the surface	Sz	34.60 µm	3.62
Skewness	Ssk	−2.70	0.31
Kurtosis	Sku	12.37	2.46

## Supplementary Materials

The following are available online at https://www.mdpi.com/1996-1944/12/6/914/s1, Figure S1: SEM/BSE images of the cross section of the coatings after pyrolysis at (a) 700 °C and (b) 800 °C (SEM/BSE). EDS-elemental distribution of (c-d) sodium, (e-f) zinc, (g-h) barium, (i-j) silicon and (k-l) zirconium., Figure S2: SEM image of the surface of the coating after pyrolysis at 800 °C in N_2_-atmosphere for 1 h, indicating closed residual porosity.
