# Supplementary material for: Oxidation Resistance and Microstructure Evaluation of a Polymer Derived Ceramic (PDC) Composite Coating Applied onto Sintered Steel
Source: Materials (Basel). 2019 Mar 19;12(6):914. doi: 10.3390/ma12060914 (PMC6470509; doi:10.3390/ma12060914)
Supplement: Supplementary file 1 [file materials-12-00914-s001.pdf]

materials

MDPI

Supplementary information

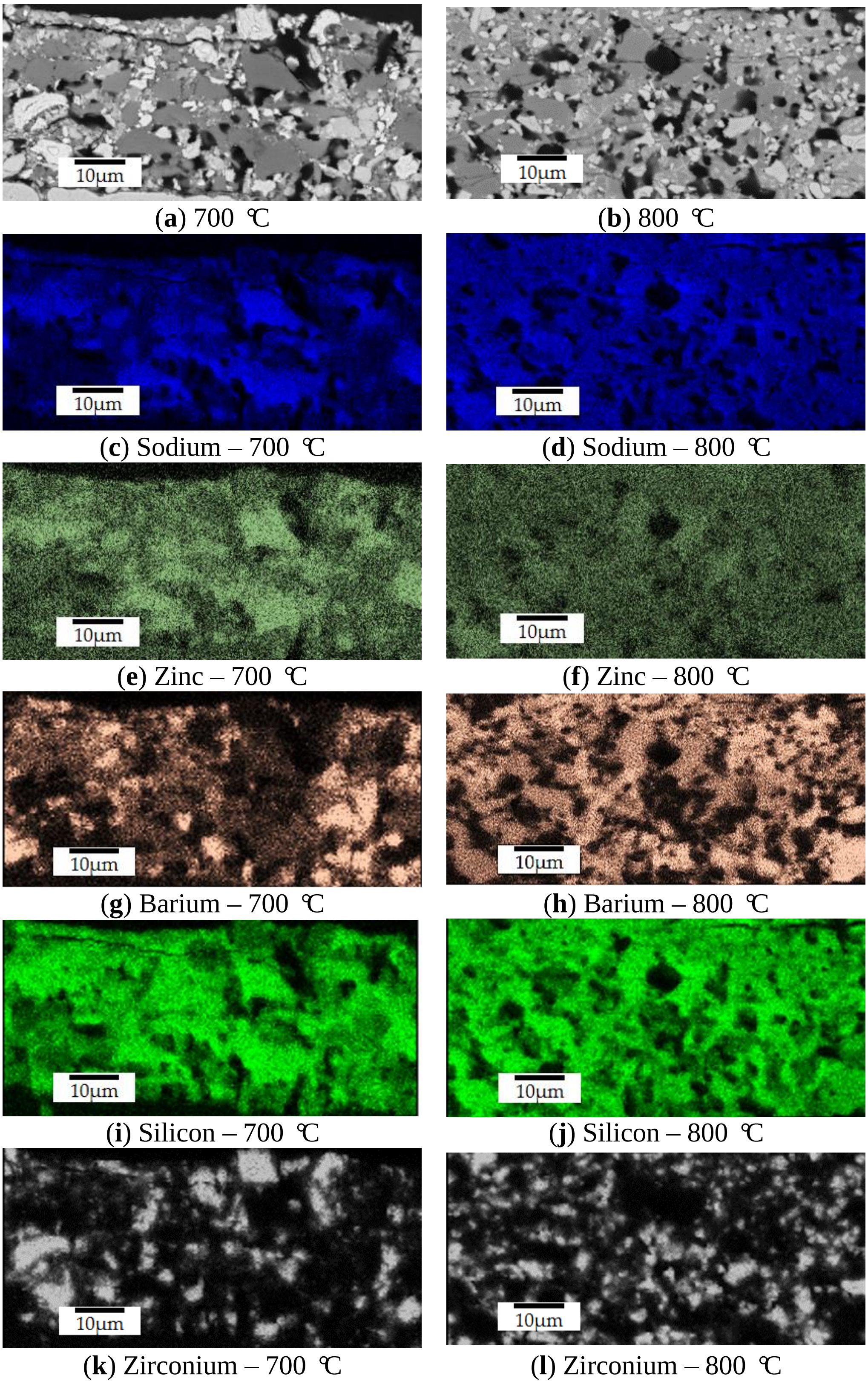

Figure S1. SEM/BSE images of the cross section of the coatings after pyrolysis at (**a**) 700°C and (**b**) 800 °C (SEM/BSE). EDS-elemental distribution of (**c-d**) sodium, (**e-f**) zinc, (**g-h**) barium, (**i-j**) silicon and (**k-l**) zirconium.

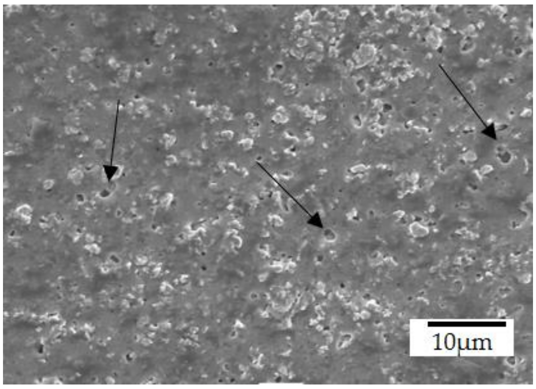

Figure S2. SEM image of the surface of the coating after pyrolysis at 800 °C in N_2-atmosphere for 1 h, indicating closed residual porosity.
